# MADAM - An open source meta-analysis toolbox for R and Bioconductor

**DOI:** 10.1186/1751-0473-5-3

**Published:** 2010-03-01

**Authors:** Karl G Kugler, Laurin AJ Mueller, Armin Graber

**Affiliations:** 1 Institute for Bioinformatics and Translational Research, UMIT, Eduard Wallnöfer-Zentrum 1, Hall in Tirol, 6060, Austria

## Abstract

**Background:**

Meta-analysis is a major theme in biomedical research. In the present paper we introduce a package for R and Bioconductor that provides useful tools for performing this type of work. One idea behind the development of MADAM was that many meta-analysis methods, which are available in R, are not able to use the capacities of parallel computing yet. In this first version, we implemented one meta-analysis method in such a parallel manner. Additionally, we provide tools for combining the results from a set of methods in an ensemble approach. Functionality for visualization of results is also provided.

**Results:**

The presented package enables the carrying out of meta-analysis either by providing functions directly or by wrapping them to existing implementations. Overall, five different meta-analysis methods are now usable through MADAM, along with another three methods for combining the corresponding results. Visualizing the results is eased by three included functions. For developing and testing meta-analysis methods, a mock up data generator is integrated.

**Conclusions:**

The use of MADAM enables a user to focus on one package, in turn enabling them to work with the same data types across a set of methods. By making use of the snow package, MADAM can be made compatible with an existing parallel computing infrastructure. MADAM is open source and freely available within CRAN http://cran.r-project.org.

## Background

R http://www.r-project.org and the associated Bioconductor [1] project have become one of the most widespread tools for bioinformatics in recent years. At approximately the same time, dozens of newly developed meta-analysis methods have been published [2-6]. For most of these methods, an implementation exists, but some still lack publicly available implementions in R. In the present paper, we present the MADAM package, which provides new implementations of two methods [5,6]. In addition, we also implemented wrappers to the existing meta-analysis methods [2,4].

Since new methods and algorithms need to be tested and validated as well, we integrated a validation model for generating simulated data. For integrating results of different meta-analysis methods using an ensemble approach, three of these methods are also implemented in MADAM. For visualizing the meta-analysis results, we implemented three further methods.

The Meta-Analysis Data Aggregation Methods (MADAM) toolbox is helpful to both developers of meta-analysis methods as well as to biologists, physicians, and bioinformaticians who conduct research across studies. Here, we present version 1.0 of the package, in turn describing a sample work flow and illustrating its use via some examples.

MADAM is available using CRAN, and can be installed using R itself:

*> install.packages("MADAM")*

After the installation it can be loaded into the current workspace:

*> library(MADAM)*

## Results

### Creation of random validation data

The implementation and validation of meta-analysis methods are eased when referring to data with known content. If the rate of true and false findings can be assessed, parameterizing and fine tuning can be facilitated more effectively. The MADAM package facilitates the creation of mock up data in a simple way. First, we create a sample set of studies that will be re-used further on in the present paper. We assume to have three studies with two experimental factors ("group 1", "group 2"), and a number of 10, 5, and 27 samples in group 1, respectively 17, 6, and 33 in the second group. Furthermore, we will only use 100 features to be simulated, for performance and demonstration reasons. We additionally need a data structure to store the class labels within, which will be referred to as cl further on. A vector cl.val contains the labels that are being used. To make the example reproducible we set a seed first.

*> set.seed(1234)*

*> gg <- 100*

*> ii <- 3*

*> kk <- c(10, 17, 5, 6, 27, 33)*

*> cl.val <- c(1, 2)*

*> A <- generateRandomMAData(g = gg, i = ii, k = kk)*

*> cl <- lapply(A, function(a) {*

*+ factor(as.numeric(a$group))*

*+ })*

*> direction <- rep("N", nrow(A[[1]]))*

*> direction [grep("_U", featureNames(A[[1]]))] <- "U"*

*> direction [grep("_D", featureNames(A[[1]]))] <- "D"*

*> table(direction)*

direction

D N U

5 90 5

The list A now contains a list of ExpressionSet objects that can be re-used for further analysis in the MADAM package. We now can also see that five features are up regulated in the second group (U) and another five are down regulated (D).

### Checking the quality of the study data

When it comes to biomedical data, one is often confronted with data thate are of imperfect quality. We included a set of methods for dealing with this problem in the present package. In the current implementation, two methods are provided in order to deal with the problems of data with no variance and missing values for a feature of interest.

Sometimes variance of a feature is zero. Such non existing variation is biological highly unlikely and might indicate problematic data. Since in standard single data set analysis features with a low variance are likely to be filtered they normally do not cause any problems. In the case of meta-analysis, where typically no such filtering is applied, these seldom events may cause problems for further analysis. Performing a t-test would result in non numeric results, since the standard error would be zero as well. We, therefore, added a method corrVar that enables a user to correct for variances of zero. For this correction, we add a vector *θ *to the feature value *α*, where *θ *~*N*(*μ*, *σ*), for each group separately. The default for *μ *is zero, and *σ *is the mean of all of the features' standard deviations within the corresponding group.

To simulate this behavior, we manually set some of the entries in A to have a variance of zero:

*> exprs(A[[1]]) [1, ] <- exprs(A[[1]])[1, 1]*

*> exprs(A[[2]]) [37, ] <- exprs(A[[2]]) [37, 1]*

*> exprs(A[[3]]) [16, ] <- exprs(A[[1]]) [16, 1]*

*> sapply(list(exprs(A[[1]]) [1, ], exprs(A[[2]]) [37, ], exprs(A[[3]]) [16, + ]), var, na.rm = TRUE)*

[1] 0 0 0

*> A <- corrVar(A, cl, cl.val)*

starting variance correction...

*> sapply(list(exprs(A[[1]]) [1, ], exprs(A[[2]]) [37, ], exprs(A[[3]]) [16, + ]), var, na.rm = TRUE)*

[1] 0.02415640 0.01546770 0.03559078

Another problem that often occurs in the data of biomedical measurements are missing values. The sources of missing values are widespread, potentially originating from measurements being below the limit of detection or due to other technical issues. Simply not having measured a single feature by an assay is another potential source for missing values. In any case, we distinguish here between all the measurements missing for a certain feature (indicating a problem of a systematic nature) or just a sparse distribution of missing values through out the whole frame of measurements. While we do not yet provide solutions for the first type of error in the MADAM package, we do provide functions for dealing with sparsely distributed missing values.

The function corrNA provides two different imputation approaches for missing values. The first method substitutes missing values up to a ratio of *ρ *within one group. For this substitution, we replace all of the missing values again by *θ*, but this time *μ *being the feature's mean value within the group, and *σ *being the standard deviation within the group. The other option for detecting features with replaceable missing values, is to allow all but a number of *ϕ *features to be missing within a group, which of course can be a rather soft criterion. For both cases, we remove the feature from further analysis if the criterion is not met. For demonstration purposes, we replace some of the values within the first ExpressionSet with missing values (detonated as NA in R).

*> exprs(A[[1]]) [1, cl[[1]] == cl.val[1]] <- NA*

*> exprs(A[[1]]) [5, cl[[1]] == cl.val[2]] <- NA*

*> exprs(A[[1]]) [3, cl[[1]] == cl.val[1]] <- NA*

*> exprs(A[[1]])[3, 1] <- 8*

*> exprs(A[[1]]) [4, cl[[1]] == cl.val[1]] <- NA*

*> exprs(A[[1]]) [4, 1:2] <- c(8, 5)*

*> exprs(A[[1]]) [7, c(1, 6, 5, 3, 12)] <- NA*

*> sum(is.na(doES(A, cl, val = cl.val, useREM = TRUE, nperm = 100)$g2down$FDR))*

[1] 5

*> A <- corrNA(A, method="madam", cl, cl.val, exclude = TRUE)*

starting NA correction...

*> res.es.g2down <- doES(A, cl, val = cl.val, useREM = TRUE, nperm = 1000)$g2down*

*> sum(is.na(res.es.g2down$FDR))*

[1] 0

As additional method for imputing values, a knn based imputation method was enabled in MADAM, as implemented in the impute package [7]. This methods imputes missing values based on information from neighbouring features. This method is selected when calling the corrNA function with the method="knn" parameter:

*> A.knn <- corrNA(A, method="knn", exclude = TRUE)*

starting NA correction...

For more details on this imputation method, see the corresponding help file in R.

### Significance based meta-analysis methods

The MADAM package contains two meta-analysis methods that are based on exploiting information about a feature's significance [5,6]. Both of these methods make use of the formula that was given by Fisher for combining p-values: , with n being the number of studies [8].

We implemented two methods for assessing the significance for *S*. The first method permutates the p-values as reported by the single studies and calculates a new  with these random combinations [6]. The ratio of  larger than *S *divided by the number of permutations than gives the p-value. Alternatively, it is possible to derive the p-value directly from *S *as it follows a *χ*^2 ^distribution with 2*n *degrees of freedom [5]. Since the first method is computationally demanding, we enabled it to be used on a cluster environment by using the snow package by Luke Tierney, which enables the carrying out of this ofthis meta-analysis in a time saving way http://www.cs.uiowa.edu/~luke/R/cluster/cluster.html. For both methods we correct for multiple testing using the FDR [9].

For deriving the p-values, we implemented a simple method for performing t-tests on a list of ExpressionSets. One can choose between the standard null hypothesis "greater", "less", or "two.sided". The resulting p-values can be stored in a matrix for later use. For replacing the p-values of zero one can give a value of choice, otherwise 1 * 10^6 ^is used.

For the presented example, we assume a cluster with 10 available nodes and running an MPI based system.

For saving time, we reduced the number of permutations.

*> clust <- makeCluster(10, type="MPI")*

   10 slaves are spawned successfully. 0 failed.

*> res.g2down <- multiTtest(A, cl, cl.val, alternative="greater")*

performing multiple tests...

[1] 1

[1] 2

[1] 3

*> res.rhodes.g2down <- doRhodesFDR(res.g2down, B = 1000, cluster = clust)*

starting method by Rhodes...

If the use of a t-test is not advised due to the small number of replicates, the user should not rely on the results from the multiTtest for this single study. In such cases the use of alternative ways, such as a moderated t-test or SAM [10], to obtain p-values is recommended. The biased p-values within the p-value storage matrix, can then be replaced with the correct ones. The implementation of a weighting schema for the significances  would be an improvement [5], and is planned to be implemented in future versions.

### Performing an ensemble approach

It is possible to combine the results from a set of meta-analysis methods in an ensemble approach. In the MADAM package, we implemented three methods for performing this task. One of the methods uses rank products: We multiply all of the ranks and divide them by the number of genes in the meta-analysis. We then shuffle the ranks again randomly and count the number of random rank products that are smaller than the expected one. Dividing this ratio by the number of permutations gives the corresponding p-value [4]. Again, this p-value was then corrected for multiple testing by using the qvalue package [9]. In the following example, we re-used the ranks reported for under expressed features in the second group as reported from the Effect Size method and one of the significance based methods. The permutation parameters are again set very low, but this is only carried out for demonstration of the methods. We re-use the cluster object that was created before, as the ensemble methods are implemented to work on such an infrastructure as well. A second ensemble method, using information on significances in order to integrate the results from the three methods, is demonstrated hereinafter.

*> res.rp.g2down <- doRP(A, cl, cl.val, nperm = 1000)$g2down*

   The data is from 3 different origins

Rank Product analysis for two-class case

Starting 1000 permutations...

Computing pfp...

*> ranks <- cbind(res.es.g2down$rank, res.rhodes.g2down$rank, res.rp.g2down$rank)*

*> pvalues <- cbind(res.es.g2down$p.value, res.rhodes.g2down$p.value, + res.rp.g2down$p.value)*

*> rownames(ranks) <- rownames(res.es.g2down)*

*> rownames(pvalues) <- rownames(res.es.g2down)*

*> res.ens.rp <- calculateRankProduct(ranks, B = 10000, cluster = clust)*

starting rank product ensemble approach

*> res.ens.fm <- fisherMethod(pvalues)*

starting Fishers method...

*> res.ens.rp [grep("_D", rownames(res.ens.rp)), ]*

      RP p.value q.value rank

ART_47_D 0.33333333 4e-04 0.0128 3

ART_60_D 0.07291667 0e+00 0.0000 1

ART_97_D 0.46875000 6e-04 0.0144 4

ART_99_D 0.25000000 1e-04 0.0048 2

*> res.ens.fm [grep("_D", rownames(res.ens.fm)), ]*

      S p.value q.value rank

ART_47_D 35.78010 3.211309e-07 7.707141e-06 4

ART_60_D 55.26204 2.863099e-11 2.748575e-09 1

ART_97_D 36.84136 1.942068e-07 6.214618e-06 3

ART_99_D 39.24931 6.187396e-08 2.969950e-06 2

In both cases, we can see that the features of interest, which are marked with "_D" are always highly ranked. There are only four entries listed, since one feature was removed during missing value correction. For performing the complete analysis, a method doEnsemble provides a shortcut interface to all of those steps mentioned above:

*> res.ens.complete <- doEnsemble(A, cl, val = cl.val, do.FM = TRUE*,

*+ perm.ES = 10, perm.RP = 10, perm.ENS.RP = 10, perm.ENS.RS = 10*,

*+ do.ENS.FM = TRUE, cluster = clust, useREM = TRUE, write.all = FALSE*,

*+ plot.fdr = FALSE, plot.ranks = FALSE)*

*> stopCluster(clust)*

When combining meta-analysis information based on the application of different methods on the same underlying data, the demonstrated Ensemble methods have to be regarded as being for exploratory use only, due to correlations within the results. In that case reported q-values do not indicate a real underlying biological significance, but are just for ranking potential features of interest for later validation.

### Visualization of the results

**Figure 1:**
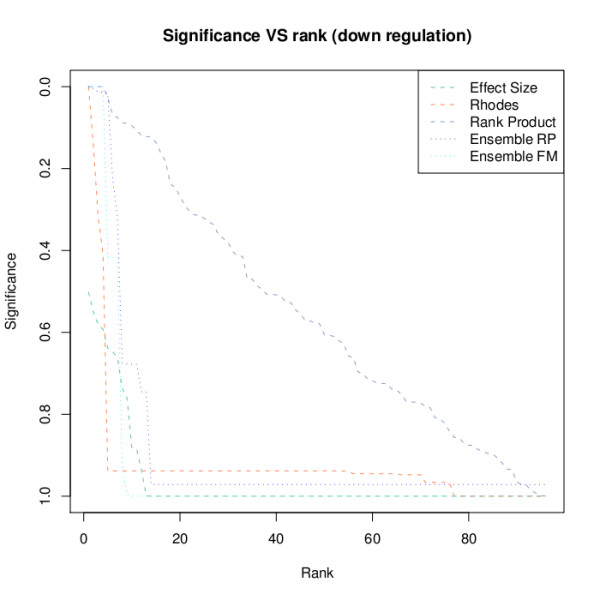
**FDR vs rank plot for under expressed features**. For visualizing the outcome of a meta-analysis, one often wants to plot the reported significances against their ranks. The function plotFDR enables this to be carried out in a simple manner, combining information from various methods. Coloring and selection of the line types is carried out automatically according to whether a reported result comes from a single study analysis, a meta-analysis, or an ensemble approach.

We integrated three methods for visualizing the results of the meta-analyses. The first one clusters the ranks of the different methods hierarchically by using euclidean distances. The second one plots significances versus the rank of the specific significance. One can provide the type of the method, by which the significances were derived, thereby changing the coloring and line types automatically as can been seen in Figure 1:'

*> signi <- cbind(Effect Size*' = *res.es.g2down$FDR, Rhodes = res.rhodes.g2down$q.value*,

*+ ***'***Rank Product*' = *res.rp.g2down$q.value*, '*Ensemble RP*' = *res.ens.rp$q.value*,

*+ Ensemble FM*' = *res.ens.fm$q.value)*

*> plotFDR(signi, title="Significance VS rank (down regulation)"*,

*+ types = c("m", "m", "m", "e", "e"))*

plotting FDR...

**Figure 2:**
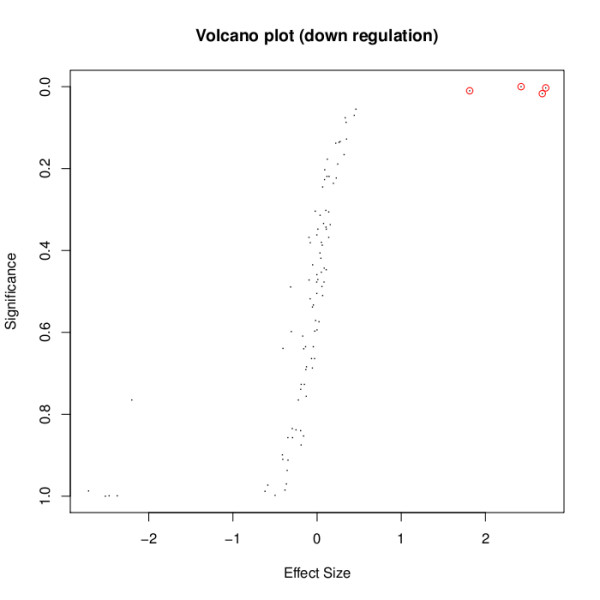
**Volcano plot for meta-analysis**. Similar to the volcano plots that are used in classical microarray analysis, the function plotMAVolcano enables the plotting of a volcano plot for meta-analysis. This is performed by plotting an effect size against a significance. Since two separate null hypotheses might be investigated in the meta-analysis, the form of the plot differs from the classical volocano-like shape. A user might also provide a list of interesting features to be highlighted automatically.

Another plotting function implemented in the MADAM package enables the plotting of a degenerated volcano plot (Figure 2). Degeneration in this context refers to the fact that the testing of two separate null hypotheses results in plots that lose their typical volcano like shape. Nevertheless, since in this plot the effect size is plotted against a significance, we still refer to it as a volcano plot:

*> volc <- data.frame(res.es.g2down$MUvals, res.rhodes.g2down$p.value*,

*+ row.names = rownames(res.es.g2down))*

*> volc.points <- volc [grep("_D", rownames(volc)), ]*

*> plotMAVolcano(volc, title="Volcano plot (down regulation)"*,

*+ points = volc.points)*

## Conclusions

The MADAM package helps those researchers in biology and medicine who utilize meta-analysis methods in their research. This package enables one to use the rich arsenal of the available methods in R, by simultaneously working with one data type only.

The possibility to use parallel computing infrastructure facilitates the efficient use of meta-analysis even more. To make use of the supercomupting abilites within MADAM, the user has to establish an infrastructure compatible to the technologies enabled in the snow package. The sample infrastructure used in this paper was based on 4 × quadcore CPUs, running on Ubuntu 9.04 with Open MPI 1.3.2.

## Outlook

We are currently planning the implementation of some other existing meta-analysis methods in a parallel manner so that they can be used in various cluster environments. Furthermore, the methods for dealing with missing values will be enhanced.

## Availability and requirements

**Package names**: MADAM

**Package version**: 1.0

**Operating system(s): **Platform independent

**License: **LGPL

**Programming language: **R

**Depends: **R (> = 2.9), Biobase (> = 2.4), snow, qvalue, RankProd, RColorBrewer, GeneMeta, gplots, genefilter, impute

**Imports: **RColorBrewer, GeneMeta, gplots, genefilter, impute

## Competing interests

The authors declare that they have no competing interests.

## Authors' contributions

KGK and LAJM implemented and validated the scripts. AG supervised the work and the statistics. KGK drafted the manuscript. All three authors participated in the writing of the paper. All authors read and approved the final manuscript.
